# Factors associated with functional status at presentation in patients with longitudinally extensive transverse myelitis

**DOI:** 10.1186/s13023-025-03999-4

**Published:** 2025-09-02

**Authors:** Nisa Vorasoot, Pilantana Saichua, Prapassara Sirikarn, Narongrit Kasemsap, Kannikar Kongbunkiat, Somsak Tiamkao, Verajit Chotmongkol, Kittisak Sawanyawisuth

**Affiliations:** 1 Division of Neurology, Department of Medicine, Faculty of Medicine, Khon Kaen University, Khon Kaen, 40002 Thailand; 2 North-Eastern Stroke Research Group, Khon Kaen University, Khon Kaen, 40002 Thailand; 3 Department of Epidemiology and Biostatistics, Faculty of Public Health, Khon Kaen University, Khon Kaen, 40002 Thailand; 4 Department of Medicine, Faculty of Medicine, Khon Kaen University, Khon Kaen, 40002 Thailand

**Keywords:** Expanded disability status scale, Clinical predictor, Cord lesion, Longitudinally extensive transverse myelitis

## Abstract

**Background:**

Longitudinally extensive transverse myelitis (LETM) is a rare neurological disease. A case series evaluated the predictors of a long-term clinical outcome. However, there is limited data on predictors of functional status at presentation. Therefore, this study aimed to find clinical factors predictive of poor clinical presentation in Asian patients with LETM.

**Methods:**

This was a cross-sectional, retrospective analytical study. The inclusion criteria were adult patients with an age of 18 years or more who met the criteria of LETM. Clinical factors of eligible patients were recorded. Eligible patients were categorized into two groups based on the Expanded Disability Status Scale (EDSS) at the time of presentation; 6 or more vs. 0-5.5. A predictive model for the EDSS score at the time of presentation was created by the logistic regression analysis.

**Results:**

There were 40 patients with LETM in the study. Of those, 31 patients (77.50%) had an EDSS of 6 or more. There were two factors in the model predictive of the EDSS at presentation of 6 or more; thoracic lesion and complete lesion. Only a complete lesion was independently associated with an EDSS at presentation of 6 or more with an adjusted odds ratio of 15.202 (95% confidence interval of 2.028, 113.968; *p* value = 0.008).

**Conclusions:**

The complete cord lesion was independently associated with severe functional status in patients with LETM at presentation.

## Introduction

Longitudinally extensive transverse myelitis (LETM), characterized by continuous spinal inflammation for at least three vertebral segments as evidenced by spinal cord magnetic resonance imaging (MRI), is a rare neurological condition but can lead to severe disability [1–3]. In the literature, most reports are either case reports, case series, or studies with small sample sizes related to several conditions [4–10]. The most common condition associated with LETM is neuromyelitis optica spectrum disorder (NMOSD), but it can be found in other conditions such as acute disseminated encephalomyelitis (ADEM), Sjogren’s syndrome, or viral infection [9].

As LETM is a rare disease, there is limited data on clinical factors predictive of a poor clinical outcome. The case series from Argentina found that patients with LETM who had spinal cord lesions of seven or more segments had a poorer functional score than those with fewer spinal cord involvements; odds ratio of 20.0 and *p*-value < 0.001 [4]. The study from China found that the causes of the non-NMOSD group of patients with LETM were mostly subacute combine degeneration and anterior spinal artery syndrome [2]. However, these two studies did not evaluate the predictors of functional status at presentation. Therefore, this study aimed to find clinical factors predictive of poor clinical presentation in Asian patients with LETM.

## Materials and methods

This was a cross-sectional, retrospective analytical study. The inclusion criteria were adult patients with an age of 18 years or more who met the criteria of LETM, defined as having abnormal spinal cord findings on the T2-weighted magnetic resonance imaging (MRI) for three or more vertebral segments. Those with syringomyelia, spinal cord trauma, or compression were excluded. Eligible patients were searched from the MRI database using the search terms of extensive cord, long myelitis, longitudinal cord, transverse myelitis, and extensive myelopathy. The study period was between January 2015 and October 2021. The study site was Srinagarind Hospital, a University Hospital of Khon Kaen University, Thailand.

Charts and MRI findings were reviewed for those who met the study criteria. Data extraction included baseline characteristics, clinical features, and the outcomes. Clinical presentations were categorized as optic neuritis, myelitis, or diencephalic, while cerebrospinal fluid (CSF) findings were also recorded for CSF white blood cell, CSF protein, and CSF glucose/plasma glucose ratio. Regarding MRI findings, levels of involvement, characteristics of axial involvement (complete, central, dorsolateral, and anterior), the number of segments involved, and Gadolinium enhancement were recorded. Serum antibodies to aquaporin-4 (AQP4) and causes of the LETM were also evaluated. The primary outcome of this study was the disability status defined by the Expanded Disability Status Scale (EDSS) at the time of presentation.

Statistical analysis. Eligible patients were categorized into two groups based on the EDSS score at the time of presentation; 6 or more vs. 0-5.5. Descriptive statistics were used to compare clinical features between both groups. Differences were assessed using inferential statistics, either Wilcoxon rank sum test for numerical variables or Fisher Exact test for categorical variables. A predictive model for the EDSS score at the time of presentation of 6 or more was executed by the univariable and multivariable logistic regression analyses. Factors in the predictive model were presented as unadjusted and adjusted odds ratios with their 95% confidence intervals and *p*-values. Hosmer-Lemeshow chi square was used to evaluate the goodness of fit of the predictive model. Statistical analysis was performed by the STATA software, version 10.0 (College Station, Texas, USA).

## Results

There were 40 patients with LETM in the study. Of those, 31 patients (77.50%) had an EDSS of 6 or more. Baseline characteristics and clinical features of the patients in both groups are shown in Table 1. Patients with LETM who had the EDSS at presentation of 6 or more had slightly older age than those who had the EDSS at presentation of 5.5 or less (55 vs. 51 years; *p* = 0.770). Regarding sex distribution, there was a comparable percentage of male sex between both groups (48.39% vs. 44.44%; *p* = 0.999). There were three significant different factors observed between both groups, including complete lesion, dorsolateral lesion, and anterior lesion (Table 1). Those with an EDSS at presentation of 6 or higher proportion of complete lesions than those who had an EDSS at presentation of 5.5 or less (93.55% vs. 44.44%; *p* = 0.003) but fewer proportions for dorsolateral and anterior lesions (0% vs. 22.22%; *p* = 0.046).

**Table 1:** Baseline characteristics and clinical features of patients with longitudinally extensive transverse myelitis categorized by an expanded disability status scale (EDSS) at presentation

Factors	EDSS 0-5.5 *n* = 9	EDSS 6-9.5 *n* = 31	*p*-value
Age, years*	51 (41–56)	55 (43–61)	0.770
Male sex	4 (44.44)	15 (48.39)	0.999
Duration of disease, years	4 (2–6)	3 (1–6)	0.873
Smoking	1 (11.11)	5 (16.13)	0.999
Clinical presentations			
Optic neuritis	2 (22.22)	1 (3.23)	0.121
Myelitis	8 (88.89)	31 (100)	0.225
Diencephalic	1 (11.11)	0	0.225
Cerebrospinal fluid (CSF) findings*			0.256
CSF white blood cells, cells/mm^3^	0 (0–55)	10 (3–55)	0.198
CSF protein, mg/dL	26 (22–62)	85 (41–186)	0.066
CSF glucose/plasma glucose, %	60 (56–69)	45 (32–57)	0.149
Magnetic resonance findings			
Level			
Cervical	7 (77.78)	12 (38.71)	0.060
Thoracic	5 (55.56)	27 (87.10)	0.059
Lumbar	1 (11.11)	9 (29.03)	0.404
Conus	0	6 (19.35)	0.306
Axial			
Complete	4 (44.44)	29 (93.55)	0.003
Central	1 (11.11)	2 (6.45)	0.545
Dorsolateral	2 (22.22)	0	0.046
Anterior	2 (22.22)	0	0.046
Number of segments*	6 (6–7)	7 (4–9)	0.472
Gadolinium enhancement	4 (44.44)	18 (60.00)	0.465
Serum AQP4IgG positive	2 (50.00)	8 (34.78)	0.613
Diagnosis			
CIS	2 (22.22)	1 (3.23)	0.121
Multiple sclerosis	0	1 (3.23)	0.999
NMOSD	3 (33.33)	12 (38.71)	0.999
ADEM	0	2 (6.45)	0.999
SLE	0	3 (9.63)	0.999
Neoplasm	0	1 (3.23)	0.999
Infection	1 (11.11)	4 (12.90)	0.999
Vascular	0	1 (3.23)	0.999
Metabolic	1 (11.11)	0	0.225
Idiopathic	1 (11.11)	3 (9.68)	0.999
SDAVF	0	2 (6.45)	0.999
Others	1 (11.11)	1 (3.23)	0.404

Note. Data presented as number (percentage); * indicated median (1st to 3rd quartile range); AQP4-IgG: Antibodies to aquaporin 4; CIS: Clinically isolated syndrome; NMOSD: Neuromyelitis optica spectrum disorder; ADEM: Acute disseminated encephalomyelitis; SLE: Systemic lupus erythematosus; SDAVF: Spinal dural arteriovenous fistulas

There were two factors in the model predictive of the EDSS at presentation of 6 or more; thoracic lesion and complete lesion (Table 2). Only a complete lesion was independently associated with an EDSS at presentation of 6 or more with an adjusted odds ratio of 15.202 (95% confidence interval of 2.028, 113.968; *p* value = 0.008). The Hosmer-Lemeshow chi square of the model was 0.25 (*p* = 0.614), indicating a goodness of fit of the model.

**Table 2:** Factors associated with an expanded disability status scale (EDSS) of 6 or more at presentation in patients with longitudinally extensive transverse myelitis by logistic regression analysis

Factors	Unadjusted odd ratio (95% confidence interval)	*p*-value	Adjusted Odd ratio (95% confidence interval)	*p*-value
Thoracic	5.400 (1.003, 29.051)	0.049	3.989 (0.540, 18.687)	0.176
Complete	18.125 (2.592, 126.721)	0.003	15.202 (2.028, 113.968)	0.008

## Discussion

Regarding the causes of LETM, this study found that NMOSD was the most common cause (37.5%), as shown in Table 1. This proportion was comparable to the findings of two previous studies from China (42.7%) and Argentina (37%) [2, 4]. These data may imply that the most common cause of LETM may not be different among both continents. However, the other causes of LETM were not prominent in this study, similar to the findings in the study from Argentina [4]. Unlike the two studies, the Chinese study found that autoimmune disease was prominent at 66.25% [2]. It is noteworthy that this study had a very high proportion of complete cord lesions at 82.5%, while the Chinese study reported a complete cord lesion rate of 51.7% [2].

Only the study from China reported that clinical presentations of LETM caused by NMOSD vs. non-NMOSD were different; postrema and cervical lesion [2]. Even though both thoracic lesion and complete cord lesion were significant in univariate logistic regression analysis, only the complete cord lesion was independently associated with poor functional status at presentation. These data may imply that complete lesion is a stronger predictor than the location of lesion. A previous study [11] also found that those with a complete cord lesion in patients with systemic lupus erythematosus had a significant longer hospital stay than a partial cord lesion (23 vs. 7 days; *p* = 0.003).

There are some limitations to this study. Firstly, the sample size was small. However, despite the small sample size, an independent predictor for LETM functional status. As this was a retrospective study, there are some possible selection and information biases. Therefore, further prospective studies are required to confirm the results of this study. Secondly, this study did not evaluate the long-term outcomes. Data of EDSS were presented as the overall score; not specific to any of the eight functional systems. Finally, some factors were not evaluated such as HLA genotype, genomic data, genetic data, or antinuclear antibodies. Not all cases were evaluated for AQP4-IgG, the indicator for neuromyelitis optica spectrum disorder.

In conclusion, complete cord lesion was independently associated with severe functional status in patients with LETM at presentation.

## Data Availability

Raw data for Table 1, and 2 are not publicly available due to individual privacy but are available from the corresponding author on reasonable request.
